# Mice infected with *M*. *tuberculosis* with *Rv0678* mutations still benefit from bedaquiline treatment

**DOI:** 10.5588/ijtldopen.23.0527

**Published:** 2024-11-01

**Authors:** N. Lounis, T. Gevers, C. Villellas, K. Andries

**Affiliations:** Infectious Diseases Department, Janssen Pharmaceuticals, Beerse, Belgium.

**Keywords:** tuberculosis, mouse models, BDQ, resistance-associated variants, minimal inhibitory concentration

Dear Editor,

Bedaquiline (BDQ) is a first-in-class ATP synthase inhibitor.^1^ It has been approved by the US Food and Drug Administration and the European Medicines Agency for the treatment of multidrug-resistant TB (MDR-TB) in combination with other anti-TB drugs. In Phase 2b clinical trials C208 and C209,^2,3^ some *Mycobacterium tuberculosis* isolates displayed resistance-associated variants (RAVs) with increased BDQ minimal inhibitory concentration (MIC) levels that were higher than the European Committee on Antimicrobial Susceptibility Testing breakpoint or the WHO critical concentration. We wanted to assess the impact of increased MICs on bacteriological response to BDQ without the confounding factor of companion drugs. Therefore, we conducted a study in which mice were infected with *M. tuberculosis* isolates that have different susceptibilities to BDQ, and treated with different doses of BDQ, to assess bacterial killing in the lungs.

Female Swiss mice were inoculated intravenously with a bacterial suspension containing approximately 10^7^ colony-forming units (CFU) of either *M. tuberculosis* H37Rv strain, *Rv0678* mutant strain, or *atp*E mutant strain. The *Rv0678* mutant strain was isolated from a BDQ untreated MDR-TB patient in the C208^2^ trial, and the *atp*E mutant was selected in vitro using the *M. tuberculosis* H37Rv strain.^1^ Mice were treated with different doses of BDQ (6, 12.5, 25 or 50 mg/kg, respectively), or moxifloxacin (MFX) as a comparator, and all drugs were given once daily, 5 days a week for 4 weeks. At Day 1 post-infection, the CFU counts in the lungs were determined by plating 10-fold dilutions of homogenised suspensions onto 7H11 plus 5% bovine serum albumin (BSA) plates, and treatment was started. The 5% BSA is added to 7H11 agar plates to prevent carry-over effects of BDQ that might result in false-negative cultures.^4^ After 4 weeks of treatment, the entire suspension prepared from each individual lung was plated without dilution on 7H11 plus 5% BSA plates containing a mixture of antibiotics and an antifungal to prevent contamination. Results of cultures were recorded after incubation at 37°C for 4 weeks.

The bactericidal effect of treatment is defined as a significant decrease in the mean number of CFU in the treated group compared to the pre-treatment value (Day 1 control group). At Day 1, the mean lung CFU count was 5.4 ± 0.2 log10, 4.6 ± 0.5 log10 and 4.3 ± 0.3 log10 CFUs in mice infected with H37Rv, for *Rv0678* mutant and *atp*E mutant strains, respectively (see Table). After 4 weeks of treatment of H37Rv-infected mice, MFX killed 2.1 log10 CFUs, whereas BDQ displayed a dose-dependent bactericidal activity, killing respectively 1.9, 3.3, 3.6 and 4.1 log10 CFUs with 6, 12, 25 and 50 mg/kg BDQ (Table). After 4 weeks of treatment of mice infected with the *Rv0678* mutant strain, MFX killed 1.9 log10 CFUs, whereas BDQ displayed a dose-dependent bactericidal activity, killing respectively 1.9, 2.7, 3.5 and 3.1 log10 CFUs with 6, 12, 25 and 50 mg/kg BDQ (Table). After 4 weeks of treatment of mice infected with the *atp*E mutant strain, MFX killed 1.5 log10 CFUs, but CFU counts in BDQ-treated groups did not decrease compared to Day 1. This suggests that BDQ up to 50 mg/kg had no bactericidal activity, but all four doses of BDQ were able to reduce CFU counts compared to late controls at Week 4, demonstrating a bacteriostatic effect (Table).

**Table.** CFU counts in the lungs of mice infected with strains with different susceptibilities to BDQ and treated with MFX or with four different dosages of BDQ for 4 weeks.*

Strains	CFU counts/lungs (log10)						
	Controls		MFX	BDQ			
	Day 1 mean ± SD	Week 4 mean ± SD	Week 4: 100 mg/kg mean ± SD	Week 4: 6 mg/kg mean ± SD	Week 4: 12 mg/kg mean ± SD	Week 4: 25 mg/kg mean ± SD	Week 4: 50 mg/kg mean ± SD
H37RV^†^	5.4 ± 0.2	6.6 ± 0.4	3.6 ± 0.5	3.5 ± 0.8	2.1 ± 0.9	1.8 ± 0.8	1.3 ± 0.3
Rv0678^‡^	4.6 ± 0.3	4.9 ± 0.1	2.7 ± 0.4	2.7 ± 0.9	1.9 ± 0.9	1.1 ± 0.0	1.5 ± 0.5
atpE^§^	4.3 ± 0.5	5.6 ± 0.2	2.8 ± 0.6	4.7 ± 0.5	4.6 ± 0.4	4.4 ± 0.4	4.0 ± 0.3

* To assess the effects of BDQ doses on CFU, a linear regression model was applied including main effects for the group and the strains and the interaction between the two.

† Wild-type strain.

‡ Guanine (G) nucleotide inserted in position 198-199 of the *Rv0678* gene which resulted in a frameshift at position 66-67 amino acid.

§ Ala63Pro in the ATP synthase (*atp*E gene).

CFU = colony-forming unit; MFX = moxifloxacin; BDQ = bedaquiline; SD = standard deviation

*M. tuberculosis* strains displaying RAVs in the *Rv0678* gene and having MICs 2–8 fold higher than those of wild-type strains have been reported in clinical studies,^2,3,5–9^ in a mouse study^10^ and in reports from several countries.^11^ If the MDR-TB treatment regimen does not include a sufficient number of effective drugs, these strains can occur within the context of individualised patient management.^12^ In a study by Almeida et al.,^13^ BDQ 25 mg/kg did not reduce CFU counts when mice were infected with *M. tuberculosis* with an *Rv0678* mutation and an MIC 8-fold higher that of H37Rv strain (0.25 vs. 0.03 μg/ml, respectively), whereas 25 mg/kg BDQ was able to kill 3 log10 CFU in the H37Rv strain infected mice. In addition, a study by Xu et al.^14^ suggests that mice infected with H37Rv with a mutation in the *Rv0678* gene require a 2-month treatment duration to achieve the same CFU decrease as seen in H37RV-infected mice treated for 1 month. This lack of activity may be explained by the difference in strains used to infect mice as strains with resistance to isoniazid (INH) with *kat*G mutations have been shown to be hyper-susceptible to BDQ.^15^

In this study, only mice infected with an *atp*E mutant displaying a high MIC of 4 μg/ml for BDQ responded less efficiently to treatment compared to mice infected with the other two strains. Both mice infected with wildtype and *Rv0678* mutant *M. tuberculosis* strains responded well to treatment with BDQ, despite the MIC of the *Rv0678* mutant strain being 16-fold higher than that of the H37Rv strain (1 vs. 0.06 μg/ml, respectively). In another mouse model study,^10^ mutations in the *Rv0678* gene resulting in low-level BDQ resistance with a moderate MIC BDQ increase (3-fold) could be overcome by increasing the BDQ dose (by 8-fold, from 6.25 mg/kg to 50 mg/kg), but did not completely overcome resistance of mutations yielding an 8-fold BDQ MIC increase.^10^ In the present study, all BDQ dosages were able to overcome the *Rv0678* BDQ resistance yielding a 16-fold BDQ MIC of the *Rv0678* mutant strain. The strains used in the three previous studies were isolated from mice infected with H37Rv strain,^10,13,14^ whereas the strain used in this study is a clinical INH- and rifampicin-resistant strain isolated from a TB patient. In the current study, mice infected with the *Rv0678* mutant strain that displayed an MIC of 1 μg/ml responded well and in a similar manner as mice infected with the H37Rv strain that displayed a BDQ MIC of 0.06 μg/ml. This was unexpected given the breakpoint of BDQ was 0.25 μg/ml in 7H11 agar. However, breakpoints in the mouse model and in MDR-TB patients are not necessarily comparable, given the differences in administered doses and drug exposures achieved. In a retrospective study in China,^16^ baseline resistance from mutations in the *Rv0678* gene had no impact on culture conversion in MDR-TB patients; this was also demonstrated in the Phase 2b clinical trials C208 & C209.^2,3,17^

In conclusion, this mouse study suggests that BDQ retains bactericidal activity against an isolate from an MDR-TB patient with the *Rv0678* gene mutation mentioned in the Table, and which is described by WHO as associated with resistance.^18^ In contrast, BDQ was only bacteriostatic against a strain with an *atp*E mutation. Further studies are needed to better understand the impact of *Rv0678* mutations on BDQ efficacy, occurring in the context of different strain backgrounds and mutations conferring resistance to INH and RMP.
